# Mapping edaphic soils' conditions to identify conservation targets for pine barren and sandplain ecosystems in New York State

**DOI:** 10.1002/ece3.9282

**Published:** 2022-09-11

**Authors:** Jeffrey D. Corbin, Emma L. Flatland

**Affiliations:** ^1^ Department of Biological Sciences Union College Schenectady New York USA

**Keywords:** biodiversity conservation, gSSURGO soils database, habitat modeling, heathlands, Karner blue butterfly, openlands, restoration, shrublands, small habitat patches, soil geomorphology

## Abstract

Small habitat patches can be important reservoirs for biodiversity, capable of hosting unique species that are largely absent from the surrounding landscape. In cases where such patches owe their existence to the presence of particular soil types or hydrologic conditions, local‐scale edaphic variables may be more effective components for models that identify patch location than regional‐scale macroclimatic variables often used in habitat and species distribution models. We modeled the edaphic soil conditions that support pine barren, sandplain, and related ecosystems in New York State with the purpose of identifying potential locations for biodiversity conservation. We quantified soil percent sand and soil depth of 156 known high‐quality remnant pine barren and sandplain ecosystems to calculate threshold soil characteristics. We then mapped all soils in the state that were at least as sandy and deep as the threshold values we calculated. The total area of our map of suitable soil conditions was over 9500 km^2^, made up of forested (57%), urban (26%), agricultural (13%), and open (4%) land covers. Our analysis nearly doubled the recognized area of barren, shrubland, and grassland habitat on deep, sandy soils in New York State. Extensive forested and even agricultural cover on these soils could also be the subject of restoration to further support the biodiversity of these unique ecosystems. The presence of extensive soils in coastal and interior New York that, with the appropriate disturbance regime, have the potential to host pine barren and sandplain ecosystems offers a new perspective on these ecosystems' distribution in the past—and about how to better align conservation and restoration to preserve the future.

## Graphical abstract

Modeling of edaphic soil conditions can reveal small habitat patches that may host unique biodiversity. Our modeling of deep, sandy soils in New York State revealed abundant soils that may host pine barren and sandplain ecosystems, and the numerous rare and threatened species they host.

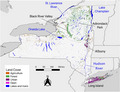

## 1 INTRODUCTION

As the scale of the biodiversity crisis becomes clear (IPBES, 2019), calls for large‐scale conservation of existing habitat have taken on renewed importance (Nicholson et al., 2019; Wilson, 2016). While much attention has been given to prioritizing large, mostly intact landscapes (Worboys et al., 2010) that avoid the known ecological traps of small or isolated patches (Murcia, 1995; Wilson et al., 2016), relatively small habitat patches are also vitally important for biodiversity conservation (Wintle et al., 2019). Such small habitat patches may be remnants of once‐larger landscapes that have been mostly lost such as old‐growth forest (Chapman et al., 2015) or grassland remnants (Stoner & Joern, 2004) and urban parks (Ives et al., 2016), or the product of edaphically unique conditions that were always patchy on the landscape such as serpentine soils (Kruckeberg, 1985), rocky outcrops (Buschke et al., 2020), and pine barrens (Motzkin & Foster, 2002). Because they differ from surrounding habitat, they may be regional or global hotspots of biodiversity, supporting species that are largely absent from the surrounding landscape or, indeed, anywhere else (Hulshof & Spasojevic, 2020; Wintle et al., 2019).

Thus, identifying, and prioritizing, opportunities to conserve small, isolated patches is of profound importance (Wintle et al., 2019). Habitat and species distribution models are useful tools for integrating climatic, geomorphic, soil, and hydrologic variables into predictions of the distribution of rare ecosystems and species (Store & Jokimäki, 2003; Williams et al., 2009). For ecosystems and species that specialize on particular soil types or hydrological conditions, local‐scale edaphic variables may be more effective predictors for patch location than regional‐scale macroclimatic variables often used in habitat and species distribution models (Velazco et al., 2017). For example, Mann et al. (1999) used soil taxonomy, geologic parent material, and rock fragment characteristics to map potential habitat of threatened limestone glades in Kentucky at both local and regional spatial scales. Likewise, Thorne et al. (2011) used maps of serpentine geology and rare species occurrences to map potential reserves in central California. Such methods can aid in identifying small patches of unique conditions that support regionally and globally significant biodiversity reserves.

**FIGURE 1:**
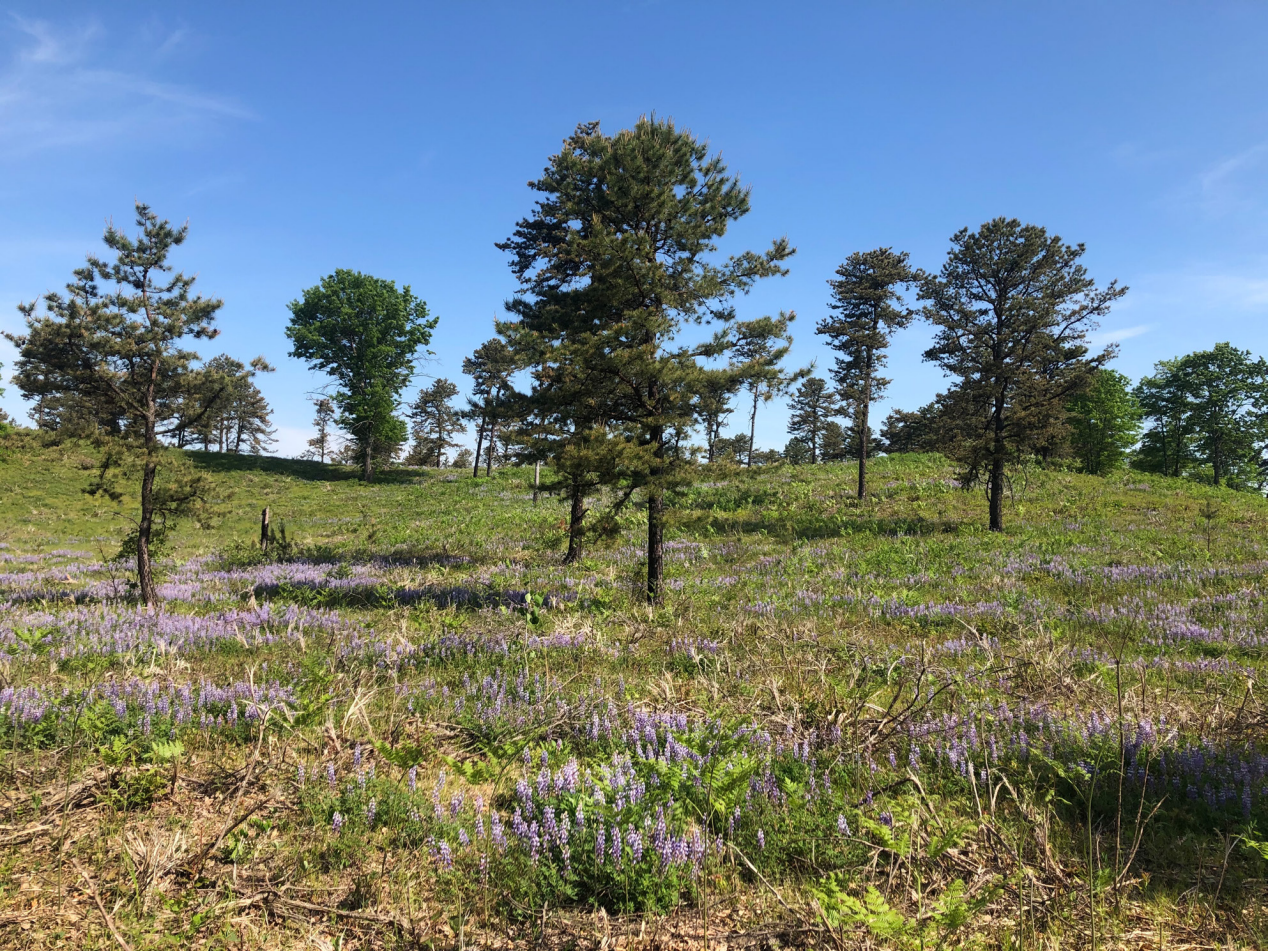
Pine barren ecosystem at Albany Pine Bush Preserve (NY). Scattered pitch pine trees are visible, with a mixed understory of perennial lupine and other herbaceous vegetation. Open sand is visible in gaps between plants.

Pine barrens, sandplains, heathlands, dunes, dwarf pine plains, and related ecosystems (hereafter referred to as pine barren and sandplain ecosystems) in the northeastern United States are an example of ecosystems that would benefit from such habitat modeling (Figure 1). They are patchily distributed across the landscape, and a variety of subtypes including pitch pine‐scrub oak barrens, coastal oak‐heath forests, dwarf pine plains, and maritime dunes are recognized as rare at the state and global level (Edinger et al., 2014). They are home to dozens of rare and threatened species including plants such as wild pink (*Silene caroliniana* ssp. *pensylvanica*), upright bindweed (*Calystegia spithamaea*), and New England blazing star (*Liatris scariosa* var. *novae‐angliae*); insects such as the frosted elfin butterfly (*Callophrys irus*) and the federally endangered Karner blue butterfly (*Plebejus melissa samuelis*); amphibians such as the eastern spadefoot toad (*Scaphiopus holbrookii*); reptiles such as the eastern hognose snake (*Heterodon platirhinos*); and birds such as the whip‐poor‐will (*Caprimulgus vociferous*), common nighthawk (*Chordeiles minor*), and the prairie warbler (*Dendroica discolor*; Albany Pine Bush Commission, 2017; New York Natural Heritage Program, 2019; Wagner et al., 2003). They are restricted to edaphically dry soil with deep layers of sand or gravel (Corbin & Thiet, 2020; Forman, 1979; Motzkin et al., 1996, 1999), but they also require frequent fires or other disturbances to prevent succession to closed‐canopy forests (Forman & Boerner, 1981; Kurczewski & Boyle, 2000; Milne, 1985; Motzkin et al., 1996). Though extensive habitat management and restoration efforts (Bried et al., 2014; Little, 1979; Pfitsch & Williams, 2009), and even the reintroduction of extirpated species (Holman & Fuller, 2011; United States Fish and Wildlife Service, 2003), are underway, intact pine barrens and sandplains occupy only a fraction of their historical area due to fire suppression and subsequent succession to forest, as well as conversion to agricultural and urban uses (Motzkin & Foster, 2002; Noss et al., 1995).

Identifying patches of pine barren and sandplain ecosystems offers the opportunity to expand conservation of these important reservoirs of biodiversity. In this paper, we used soil geomorphological variables to model the locations of conditions that support pine barren and sandplain ecosystems in New York State (USA). We analyzed the soil characteristics of known remnants of these ecosystems and extrapolated those characteristics to the rest of the state. We also quantified the current land cover of these potential areas to further narrow conservation targets and to gauge the barriers to successfully restoring biodiversity and ecosystem function. The result was a map that nearly doubled the known area of open barren, shrubland, and grassland cover on suitable soils, while also identifying abundant forest, agriculture, and urban land cover on these soils. We argue that our map can be used to identify opportunities to augment existing, conserved pine barren and sandplain ecosystems in previously overlooked areas for the benefit of the variety of rare and threatened species they support.

## 2 METHODS

We selected 27 ecosystem types identified by the New York Natural Heritage Program (NYNHP) that occur primarily on deep sandy soils (Table 1). We did not include ecosystems such as dwarf pine ridges or limestone and sandstone pavement barrens that share many characteristics and species with those in Table 1, but whose thin soils limit tree establishment and autogenic succession to hardwood forest. We mapped 156 known locations of these focus ecosystem types using data from the NYNHP (Edinger et al., 2014; New York Natural Heritage Program, 2021). We used the United States Geological Survey's Gridded Soil Survey Geographic database (gSSURGO; Soil Survey Staff, 2021; 10 m resolution) to characterize the mean percent sand and soil depth (cm) of each of these 156 pine barren and sandplain ecosystem locations. gSSURGO is a field‐validated dataset in the form of a series of geospatial polygons derived from a landscape's soil taxonomy. We did not field validate our map's predicted soil characteristics, instead relying on the gSSURGO database's robustness at the scale of our investigation (Soil Survey Staff, 2017).

**TABLE 1:** Ecosystems that occur, primarily, on deep, sandy soils, as identified by the New York Natural Heritage Program (New York Natural Heritage Program, 2021), and the areal extent in New York State.

Ecosystem	New York Area (km^2^)
Boreal heath barrens	9.0
Coastal oak‐beech forest	2.8
Coastal oak‐heath forest	19.8
Coastal oak‐hickory forest	6.3
Coastal oak‐holly forest	1.3
Coastal oak‐laurel forest	1.3
Dwarf pine plains	5.6
Great Lakes dunes	2.9
Hempstead Plains grassland	<0.1
Maritime beach	10.8
Maritime beech forest	0.3
Maritime dunes	9.3
Maritime freshwater interdunal swales	1.3
Maritime grassland	0.6
Maritime heathland	1.7
Maritime holly forest	<0.1
Maritime oak forest	3.5
Maritime pitch pine dune woodland	3.1
Maritime red cedar forest	0.3
Maritime shrubland	4.1
Pitch pine‐heath barrens	16.4
Pitch pine‐oak forest	133.2
Pitch pine‐oak‐heath woodland	50.1
Pitch pine‐scrub oak barrens	37.8
Successional blueberry heath	11.2
Successional maritime forest	2.4
Successional northern sandplain grassland	17.2
Total area	353

We characterized the mean percent sand and soil depth (cm) of the 156 ecosystem locations by, first, calculating the mean percent sand of the entire soil profile (weighted by the length (cm) of each horizon layer, Equation 1) and the depth to the nearest restrictive layer (e.g., bedrock), up to a maximum reported depth of 200 m, of each soil type that occurred within each location. Next, because each location included multiple soil types, we calculated one mean percent sand and soil depth for each location by weighting the values of the constituent soil types by their area within a location (Equations 2 and 3).

(1) $$\text{Soil type mean percent sand}=\frac{\sum_{g=1}^{m}\left[{\text{Length}}_{g}*{\text{PercentSand}}_{g}\right]}{\sum_{g=1}^{m}{\text{Length}}_{g}}$$

where Length_*g*_ is the length (cm) of each horizon, PercentSand_*g*_ is the percent sand of each horizon (g), and *m* is the number of horizons in each soil type.

(2) $$\text{Location mean percent sand}=\frac{\sum_{h=1}^{n}\left[{\text{Area}}_{h}*{\text{PercentSand}}_{h}\right]}{\sum_{h=1}^{n}{\text{Area}}_{h}}\;$$

(3) $$\text{Location mean soil depth}=\frac{\sum_{i=1}^{n}\left[{\text{Area}}_{i}*{\text{SoilDepth}}_{i}\right]}{\sum_{i=1}^{n}{\text{Area}}_{i}}$$

where Area_*h*_ and Area_*i*_ are the areas of each soil type, PercentSand_*h*_ is the mean percent sand of each soil type, calculated in Equation 1, SoilDepth_*i*_ is the depth to the nearest restrictive layer of each soil type, and *n* is the number of soil types in each location.

We established threshold values for sand content and depth that would accurately represent the typical soil characteristics of the focus ecosystems by randomly selecting 109 of the 156 locations (=70%) and calculating the area‐weighted mean for percent sand and soil depth (Equations 4 and 5).

(4) $$\text{Statewide mean percent sand}=\frac{\sum_{j=1}^{109}\left[{\text{Area}}_{j}*{\text{PercentSand}}_{j}\right]}{\sum_{j=1}^{109}{\text{Area}}_{j}}$$

(5) $$\text{Statewide mean soil depth}=\frac{\sum_{k=1}^{109}\;\left[{\text{Area}}_{k}*{\text{SoilDepth}}_{k}\right]\;}{\sum_{k=1}^{109}{\text{Area}}_{k}}\;$$

where Area_*j*_ and Area_*k*_ are the areas of each of the 109 randomly selected location, PercentSand_*j*_ is the mean percent sand of each location, calculated in Equation 2, and SoilDepth_*k*_ is the depth to the mean distance to nearest restrictive layer of each location, calculated in Equation 3. The remaining 47 locations (=30%) were used to validate our model (see below).

**FIGURE 2:**
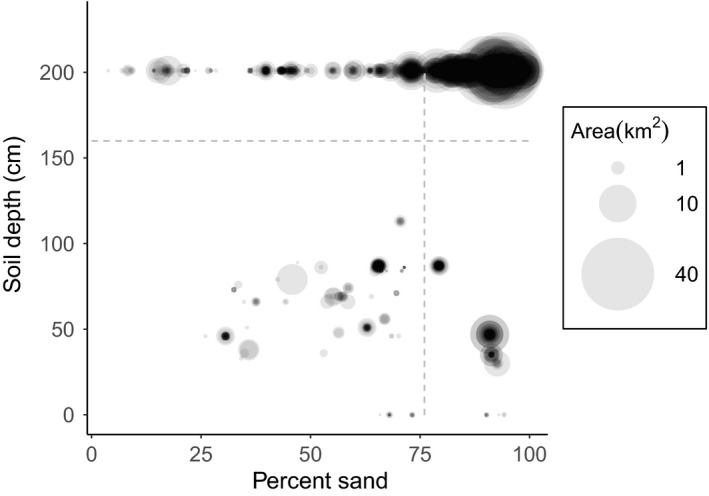
Mean percent sand (Equation 2) and depth (up to 200 cm) to the nearest restrictive layer (Equation 3) for soils within the 156 locations of focus ecosystems identified by NYNHP (New York Natural Heritage Program, 2021). Each element's symbol is scaled by its area. Circles are layered on top of each other so that combinations of percent sand and soil depth that occur at many locations appear darker. The dotted lines indicate the state‐wide threshold values of 76% sand and 160 cm depth, as determined from the area‐weighted mean percent sand and depth (Equations 4 and 5) extended to include one area‐weighted SD below the mean of 109 randomly selected locations. Those threshold values formed the basis of our model that we applied to the gSSURGO database of soil properties in New York (Soil Survey Staff, 2021).

The area‐weighted mean (± area‐weighted SD) percent sand content of the subset of these locations that we used to train our model was 87 ± 11%; the area‐weighted mean depth (± area‐weighted SD) to a restrictive layer was 193 ± 33 cm (Figure 2). We used the area‐weighted means for percent sand and depth extended to include one area‐weighted SD below the mean—at least 76% sand and at least 160 cm depth—as thresholds to define soils most likely to support pine barren and sandplain ecosystems. We applied them to the statewide gSSURGO dataset to create a map of New York's soils where mean percent sand (Equation 1) and depth to nearest restrictive layer were higher than the threshold values. We omitted areas whose land cover was wetlands or open water. The final result was a map of areas in New York where soils are suitably sandy and deep to support pine barren and sandplain ecosystems.

We validated that our modeled locations of deep sandy soils accurately represented conditions that favor pine barren and sandplain ecosystems, and their associated biota, in three ways (Appendix A). First, we calculated the proportion of the 47 focus ecosystem locations that were not used to generate threshold values (i.e., the remaining 30% of the 156 NYNHP ecosystem locations) that fell within our map of the state's deep sandy soils (Appendix A Table A1). Second, we tested whether our model avoided conditions that support ecosystems outside our focus ecosystem types by calculating the proportions of areas of the other 147 other native ecosystem types mapped by NYNHP that occurred within our map (Appendix A Table A1). Finally, we assessed the ability of our model to characterize the location of rare plants and animals that occupy pine barren and sandplain ecosystems using location maps for 58 moths and butterflies, one toad, and five plants that have close affinity to the focus ecosystems (Appendix A Table A2). Most of these species are classified as rare or species of conservation concern at the federal or state level. Sighting dates for plants and animals, as well as the dates of most recent observations of the community data, ranged from 1978 to 2017 (New York Natural Heritage Program, 2019). Most locations were identified as spatial coordinates, though some coordinates were estimated from location names (e.g., a park where the species was sighted) using GoogleEarth coordinates. We calculated the proportion of the known location of each species that intersected with our map of deep sandy soils.

In order to understand the current conditions of the soils our model identified, we intersected our map with a map of United States land cover (2019 Landsat, 30 m resolution; Yang et al., 2018). We considered four main land cover categories: forests (including needleleaf, broadleaf deciduous, and mixed); open (including shrublands, grasslands, and barrens); agriculture; and urban. We also calculated the proportion area (km^2^) of each land cover category in the entire state. Forests are defined by areas where trees (more than 5 m tall) make up at least 20% of the total vegetation; shrublands are areas where shrubs (less than 5 m tall) make up at least 20% of the total vegetation; grasslands are areas where graminoid or herbaceous vegetation makes up at least 80% of the total vegetation; barrens are areas where vegetation makes up less 15% of total cover; and agriculture includes both pasture/hay and cultivated crops. The pine barren and sandplain ecosystems that we focused on are most likely to be classified as “open” shrublands, grasslands, or barrens, but some may also have enough pine cover to be classified as forests.

We performed all spatial analysis using ArcMap (10.8.1, ESRI) and data summaries using R (R Core Team, 2022).

## 3 RESULTS

**FIGURE 3:**
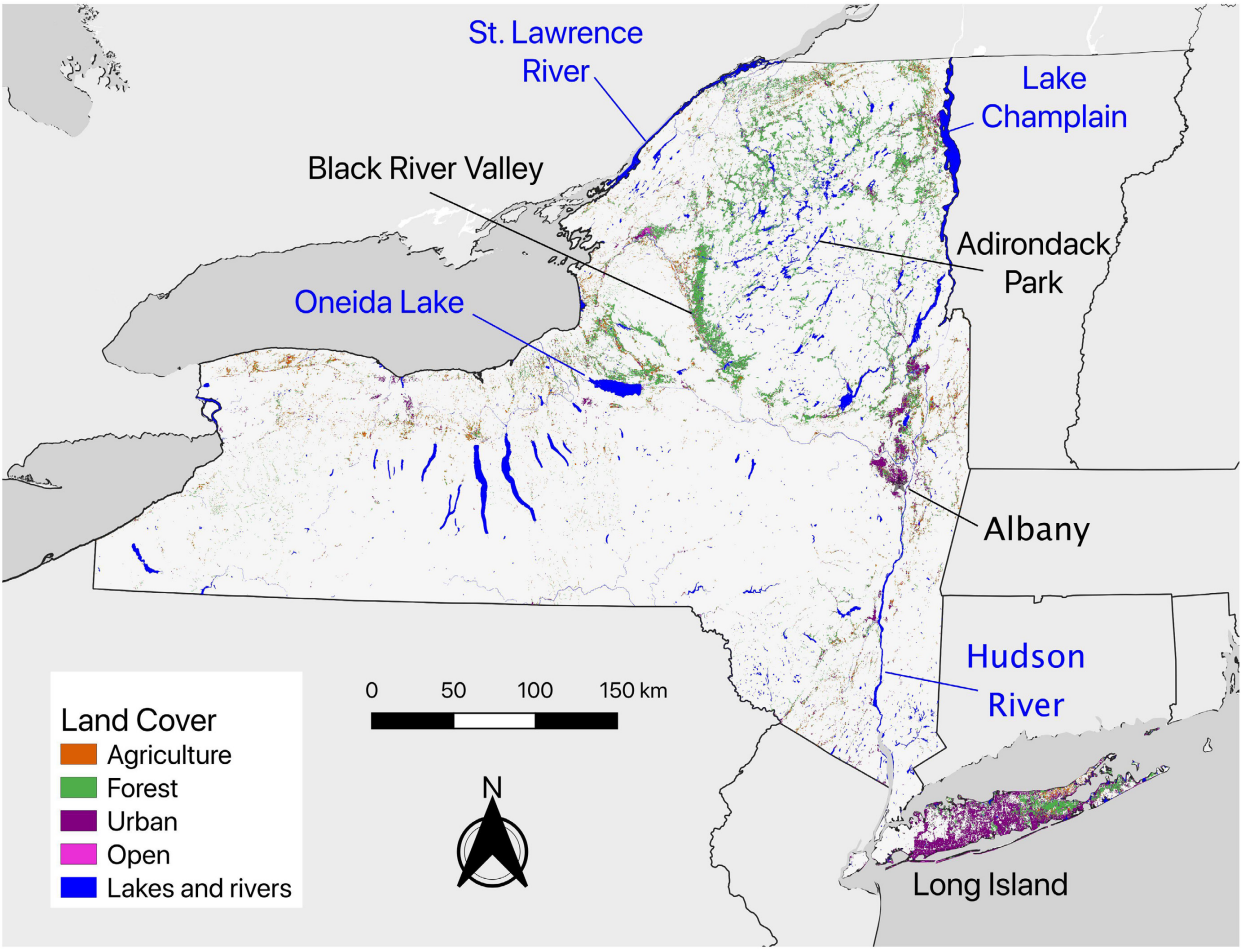
Map of soils in New York State with a depth‐weighted percent sand content of at least 76% and a depth of at least 160 cm. Current land cover (agriculture, forest, urban, and open barrens, grasslands, and shrublands) on modeled soils is indicated by color (2019 Landsat, 30 m resolution; Yang et al., 2018).

The known area of the focus ecosystems, namely those that occur primarily on well‐drained, sandy soils, identified by the New York Natural Heritage Program was 353 km^2^, or less than 0.3% of the state's terrestrial area. Our model identified 9578 km^2^ of soils that were at least as sandy and deep as our threshold values—almost 8% of the state's terrestrial area (Figure 3; Corbin & Flatland, 2021), including 319 km^2^ of additional barren, shrubland, and grassland land cover outside of known NYNHP locations. The most common present‐day land cover type on deep, sandy soils of New York is forests (57%), particularly deciduous forest. Urban (26%) and agriculture (13%) features made up most of the remaining area. Taken together, there is nearly 7000 km^2^ of forested, agricultural, and open land on deep sandy soils in New York, nearly 20 times the area of known, high‐quality remnant ecosystems. More than 60% of Long Island contained such soils. Other prominent sand elements were found near Albany, in the North Country from the northern Adirondack Park to the Canadian border, in the Black River Valley, and north of Oneida Lake (Figure 3). Each of these latter locations is associated with glacial lakes that are known to have deposited sand and gravel ~13,000 years ago.

**FIGURE 4:**
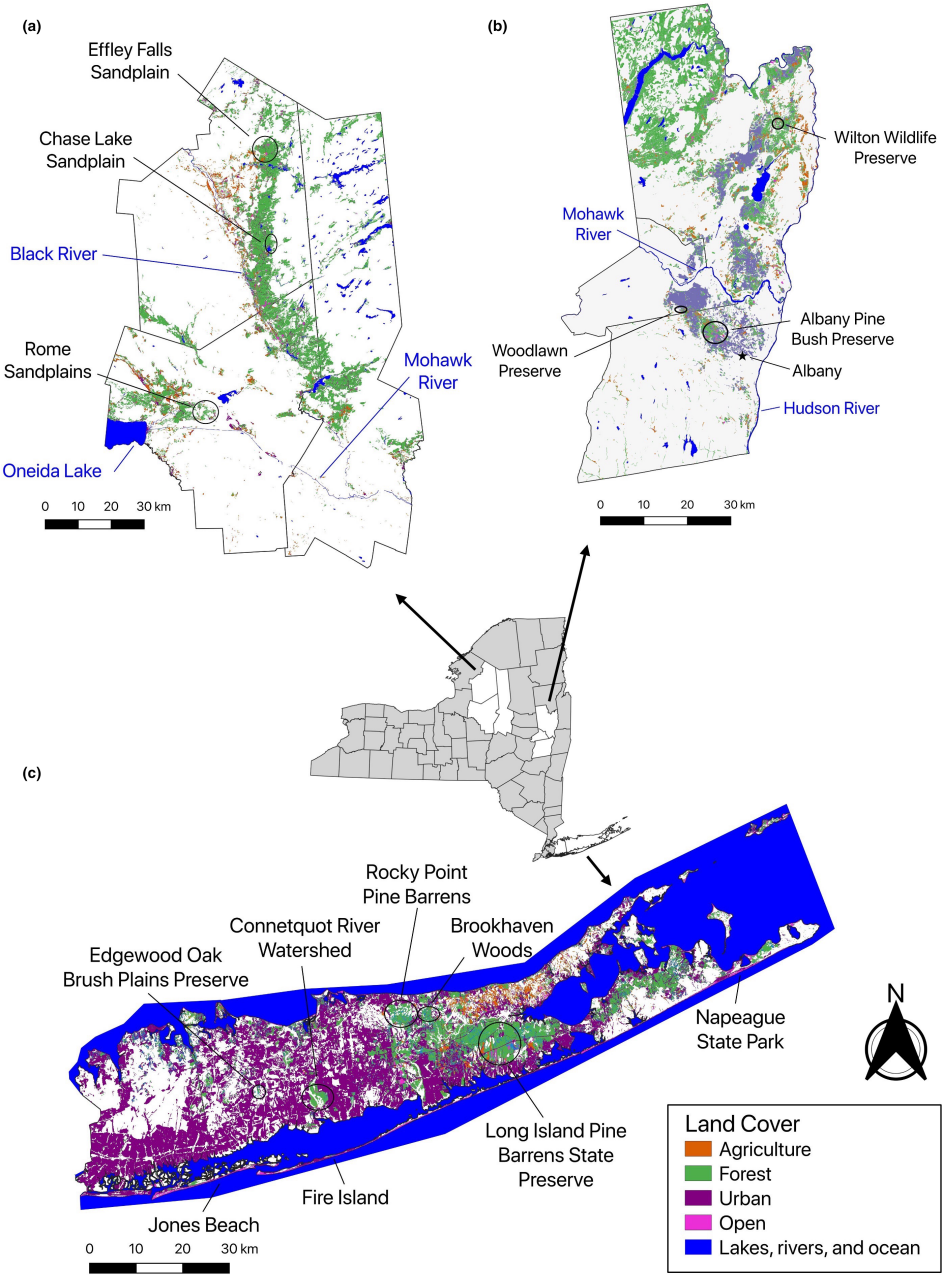
Local distribution of deep sandy soils derived from our model in select counties: (a) Herkimer, Lewis, and Oneida Counties; (b) Albany, Schenectady, and Saratoga Counties; (c) Nassau and Suffolk Counties. Select remnant pine barren, sandplain, and other focus ecosystems are indicated on each region's map.

In order to understand the distribution of deep sandy soils at the local scale where conservation and restoration planning often occurs, we examined mapped soils in three regions where our model identified extensive areas (Figure 4). Our model expanded upon the area of known pine barren and sandplain locations in all three regions—the area of barren, grassland, or shrubland on deep, sandy soils in Central New York's Herkimer, Lewis, and Oneida Counties was seven times the area identified by NYNHP; twice the area in the Capital Region's Albany, Saratoga, and Schenectady Counties; and 30% more area in Long Island's Nassau and Suffolk Counties. There were also extensive deep, sandy soils with other land covers in each region. In Herkimer, Lewis, and Oneida Counties, 79% of the deep sandy soil was forested, and 10% was agriculture (Figure 4a). The area of urban (7%) and open barren, shrubland, and grassland (4%) land covers was relatively small. The Capital District counties of Albany, Schenectady, and Saratoga were relatively evenly split between urban (47%) and forested (42%) land cover (Figure 4b). Only 2% of the area in those counties was made up of open barren, shrubland, and grassland land cover. Finally, the mapped soils in Long Island's Nassau and Suffolk Counties were mostly urban (67%), followed by forested (27%) land cover (Figure 4c). Despite the existence of several remnant pine barren and sandplain ecosystems in parks and preserves on Long Island, only 3% of deep, sandy soils, there was open land cover.

Our map coincided with the known populations of animal and plant species that have an affinity for the open, sandy ecosystems we targeted. Our map overlapped with 83% of the moth and butterfly locations identified by NYNHP (Appendix A Table A2). For the 29 moths and butterflies whose affinity to the focal ecosystems is high, the overlap was 87%. These species include the federally endangered Karner blue butterfly (98%), the state threatened frosted elfin butterfly (91%), the state species of special concern coastal barrens buckmoth (*Hemileuca maia* ssp. *5*) (87%), and a variety of other species of high conservation concern (Appendix A Table A2). The occurrences of the one vertebrate for which there was data, the eastern spadefoot toad, was also well described by the soils (79%). One plant species monitored by NYNHP that had a high affinity to barren ecosystems, Schweinitz's flat sedge (*Cyperus schweinitzii*), had a percent overlap of 80%; the other three monitored plants' overlap ranged from 38% to 72%.

## 4 DISCUSSION

Pine barren and sandplain ecosystems in New York and the rest of the northeastern United States host an assemblage of unique plants and animals of significance for regional and global biodiversity. Because their distribution is so closely tied to edaphic soil conditions, ecosystem modeling offered an opportunity to identify additional locations that might be suitable habitat—in their present state or in a restoration context. Our modeling revealed extensive areas in New York State whose soil conditions match those of existing pine barren and sandplain ecosystems. We identified nearly twice the area of barren, shrubland, and grassland as the area presently recognized by the New York Natural Heritage Program. The area that is currently open land cover comprises, nearly universally, small habitat patches; however, such small patches can be important biodiversity reservoirs when they are the product of edaphic conditions that support unique species (Velazco et al., 2017; Wintle et al., 2019).

It is notable how little of the deep, sandy soil in the state currently supports the open canopy that is likely to host the endemic biodiversity of pine barrens and sandplain ecosystems.

Urban development and agriculture have consumed nearly 40% of the area of these deep, sandy soils, and most of the remaining soils are forested. Still, the area of forested, agricultural, and open land that has the greatest restoration potential is almost 20 times the area of the high‐quality pine barren and sand plain ecosystems identified by the NYNHP. Existing examples of these pine barren and sand plain ecosystems—in the three regions we examined in detail and elsewhere—were mostly embedded within much larger matrices of forest, agriculture, and urban land cover that shared the distinctive deep sandy soils. These larger matrices, found throughout the state, offer opportunities to restore deep, sandy soils to open‐canopied conditions that support these unique ecosystems and the rare and vulnerable plants and animals they host.

A variety of projects in New York and surrounding states have successfully applied such management tools as removing tree cover, managing disturbances through mechanical harvest, fire, and selective grazing, and reintroducing key plant and animal species (Albany Pine Bush Commission, 2017; Beattie et al., 2017; Bried et al., 2015; Malcolm et al., 2008; Pfitsch & Williams, 2009; B. Hawthorne, personal communication). For example, removal of white pine trees at Rome Sand Plains boosted the populations of wild blue lupine plants and the threatened frosted elfin butterfly (Pfitsch & Williams, 2009). Similarly, the Albany Pine Bush Preserve Commission has greatly expanded pine barren habitat and population sizes of the endangered Karner blue butterfly by removing hardwood trees and reintroducing fire (Albany Pine Bush Commission, 2017; Bried et al., 2015; Gifford et al., 2020) and prescribed fire and brush cutting has enabled the successful reintroduction of the Karner blue to the Concord (NH) Pine Barrens (Holman & Fuller, 2011).

The same glacial processes that produced extensive deposits of sand and gravel in coastal and inland New York occurred elsewhere in the US Northeast and Midwest. Those soils also support pine barren and sandplain ecosystems that host unique plants and animals. The largest remaining pine barren ecosystem in North America is in New Jersey's pinelands, but similar ecosystems can also be found on Cape Cod and other coastal beaches and barrier islands of the Atlantic coast (Corbin & Thiet, 2020; Forman, 1979; Foster & Motzkin, 2003). Widely scattered, inland sand deposits from glacial lakes also support pine barren and sandplain ecosystems in Connecticut, Maine, New Hampshire, Vermont (Corbin & Thiet, 2020; Motzkin et al., 1996) and the upper Midwestern US (Radeloff et al., 1999). Modeling of deep sandy soils as potential open‐canopy habitat in these other regions has the potential to suggest further opportunities to augment current protected area for the benefit of biodiversity.

Other ecosystems besides those that occur on deep, sandy soils are likely predictable from soil conditions for the purposes of identifying potential conservation and restoration targets (Velazco et al., 2017). Pine barrens and open grasslands in New York and elsewhere in the region also occur on the edaphically thin soils of rocky slopes, summits, and limestone and sandstone plains (New York Natural Heritage Program, 2021). Such globally and regionally rare communities as dwarf pine ridges, sandstone pavement barrens, alvar grasslands and woodlands, and calcareous red cedar barrens host a similar suite of unique plants and animals as the communities that we have previously detailed. Opportunities exist to extend the modeling of edaphic conditions to identify conservation targets for the ecosystems that are restricted to these thin soils (e.g. Manitoba Alvar Initiative, 2012). Serpentine and limestone glade ecosystems, are also restricted to narrow, edaphic, soil conditions (Belcher et al., 1992; DeSelm, 1986; Kruckeberg, 1985; Proctor & Woodell, 1975), each product of specialized geology that creates unique chemical or physical soil conditions. In such cases, soil classification and soil survey data that identify the geologic conditions that drive ecosystem occurrence may be sufficient to build accurate models for potential habitat (Mann et al., 1999; Thorne et al., 2011).

### 4.1 Implications for the natural history of New York's pine barrens and sandplains

Our analysis does not reveal the distribution of pine barrens and sandplains in times past, but reconstructions from historical maps and aerial photographs in Long Island and Massachusetts reveal extensive pine barren and sandplain ecosystems in the years following European settlement (Foster & Motzkin, 2003; Jordan et al., 2003; Motzkin et al., 1996, 1999). Motzkin et al. (1999), for example, found that pinelands existed in over one‐quarter of the outwash sand deposits in Massachusetts' Connecticut River Valley. Thus, it is likely that, in the past, the area of pine barrens and sandplain ecosystems in New York State was significantly larger than the several hundred square kilometers they occupy today. Widespread fire suppression and the abandonment of Colonial‐era agricultural practices in the 19th and 20th centuries likely initiated succession to closed‐canopy forest throughout the region (Foster & Motzkin, 2003; Motzkin et al., 1999; Radeloff et al., 2000). Such forests, which now occupy a majority of deep, sandy soils in New York, are often unable to support the unique and rare species that are characteristic of pine barren and sandplain ecosystems.

Vegetation types on these soils can be quite dynamic over decadal time periods (Foster & Motzkin, 2003; Motzkin et al., 1996, 1999). For example, Motzkin et al. (1996) found wide variation in plant cover over time—from grasslands to shrub heath to sparse‐canopy pinelands to hardwood forest and back—that shifted dramatically from pre‐Colonial times to present. Viewed from this perspective, pine barren and sandplain ecosystems likely coexisted with forests within a dynamic mosaic (sensu Fuhlendorf & Engle, 2004; Wu & Loucks, 1995) that varied in space and time. A variety of ecosystem types, from grasslands or heathlands to pine or hardwood forests, were likely distributed across the state and elsewhere in the region, depending on local disturbance patterns—patterns that changed over time as well as space. However, even assuming that open‐canopy ecosystems occupied only a fraction of available soils, our map may explain how populations of the Karner blue butterfly—whose range, today, has a 1000 km gap between western Michigan and eastern New York (United States Fish and Wildlife Service, 2003)—and other open ecosystem endemics were connected in the past: hundreds of open‐canopy ecosystem patches, each occurring within several kilometers of others, would make a continuous and connected landscape that could have supported metapopulation dynamics. Any one patch could have alternated between conditions that were suitable and unsuitable for endemic species' occupancy, depending on disturbances and succession, but collectively could support a continuous metapopulation. In this way, the distribution and population dynamics of pine barren and sandplain endemics could have resembled those of serpentine endemics, whose populations are supported by a network of connected patches that form dynamic metapopulations (Harrison, 2011; Harrison et al., 1988; Kruckeberg, 1985).

## AUTHOR CONTRIBUTIONS

**Jeffrey D. Corbin:** Conceptualization (lead); formal analysis (lead); investigation (equal); methodology (equal); writing – original draft (lead); writing – review and editing (lead). **Emma L. Flatland:** Formal analysis (supporting); investigation (equal); methodology (equal); writing – review and editing (supporting).

## CONFLICT OF INTEREST

The authors declare no conflicts of interest.

## Data Availability

A GeoTIFF file of the soils we displayed in Figures 3 and 4 is available at https://doi.org/10.6084/m9.figshare.17702561.v1.

## APPENDIX A

We validated that our modeled locations of deep sandy soils accurately represented conditions that favor pine barren and sand plain ecosystems by calculating the proportion of the 47 focus ecosystem locations that were not used to generate threshold values (i.e., the remaining 30% of the 156 NYNHP ecosystem locations) that fell within our map of the state's deep sandy soils. Our map coincided with 81% of the area of those ecosystems. Among key ecosystems that matched closely were pitch pine‐oak forest (89% of their area was within mapped soils), pitch pine‐scrub oak barrens (85%), and pitch pine‐oak‐heath woodlands (84%; Table A1). Maritime beach (86%), coastal oak‐heath forest (83%), and boreal heath barrens (82%) also mostly coincided with mapped soils. Ecosystems that matched moderately closely included successional northern sandplain grassland (65%) and coastal oak‐hickory forest (65%). Focus ecosystems for which less than 50% of their area coincided with our map, such as coastal oak‐holly forest, Great Lakes dunes, and maritime oak forest, were relatively limited (<5 km^2^) in their known extent (Table A1).

**TABLE A1:** The area of all rare or high‐quality native ecosystems as recorded by the New York Natural Heritage Program (NYNHP) data (New York Natural Heritage Program, 2021) and the percentage of that area that occurs on soils identified by our soil model.

Community	NYNHP Area (km^2^)	Percentage match with soil model
Acidic talus slope woodland	6.1	<1
Allegheny oak forest	24.3	0
Alpine krummholz	4.2	0
Alpine sliding fen	<0.1	0
Alvar pavement grassland	20.4	<1
Alvar woodland	16.1	<1
Appalachian oak‐hickory forest	212.8	<1
Appalachian oak‐pine forest	38.4	7
Aquatic cave community	<0.1	0
Backwater slough	1.5	<1
Balsam flats	40.4	14
Beech‐maple mesic forest	1,977.5	<1
Black spruce‐tamarack bog	75.3	6
Bog lake/pond	<0.1	0
*Boreal heath barrens*	*8.9*	*82*
Brackish interdunal swales	1.0	45
Brackish intertidal mudflats	2.0	<1
Brackish intertidal shore	<0.1	79
Brackish meadow	0.4	41
Brackish subtidal aquatic bed	2.3	0
Brackish tidal marsh	3.3	<1
Calcareous cliff community	5.3	<1
Calcareous pavement woodland	0.6	1
Calcareous red cedar barrens	<0.1	0
Calcareous shoreline outcrop	9.8	1
Calcareous talus slope woodland	7.9	0
Chestnut oak forest	669.1	<1
Cliff community	2.1	9
*Coastal oak‐beech forest*	*2.8*	*72*
*Coastal oak‐heath forest*	*19.8*	*83*
*Coastal oak‐hickory forest*	*6.3*	*65*
*Coastal oak‐holly forest*	*1.3*	*8*
*Coastal oak‐laurel forest*	*1.3*	*67*
Coastal plain Atlantic white cedar swamp	0.3	14
Coastal plain pond	0.3	6
Coastal plain pond shore	2.0	16
Coastal plain poor fen	0.2	4
Coastal salt pond	1.1	2
Cobble shore	0.6	6.5
Cobble shore wet meadow	0.5	2
Confined river	10.3	6
Deep emergent marsh	168.9	<1
Dry alvar grassland	0.3	0
*Dwarf pine plains*	*5.6*	*96*
Dwarf pine ridges	6.8	0
Dwarf shrub bog	14.2	2
Eutrophic dimictic lake	0.3	1
Eutrophic pond	0.6	0
Floodplain forest	129.2	2
Floodplain grassland	0.1	8
Freshwater intertidal mudflats	3.3	<1
Freshwater intertidal shore	0.2	1
Freshwater tidal creek	<0.1	0
Freshwater tidal marsh	6.6	<1
Freshwater tidal swamp	3.3	<1
Great Lakes aquatic bed	25.0	<1
Great Lakes bluff	0.2	3
*Great Lakes dunes*	*2.9*	*43*
Great Lakes exposed shoal	7.2	0
Hemlock‐hardwood swamp	7.4	5
Hemlock‐northern hardwood forest	547.0	2
*Hempstead Plains grassland*	*<0.1*	*25*
High salt marsh	46.2	1
Highbush blueberry bog thicket	3.0	3
Ice cave talus community	4.7	23
Inland Atlantic white cedar swamp	0.6	0
Inland calcareous lake shore	0.2	7
Inland noncalcareous lake shore	0.6	16
Inland poor fen	7.6	5
Inland salt marsh	<0.1	<0
Inland salt pond	1.4	0
Intermittent stream	0.2	0
Limestone woodland	49.5	7
Low salt marsh	42.0	2
Maple‐basswood‐rich mesic forest	120.6	2
Marine back‐barrier lagoon	351.6	9
Marine eelgrass meadow	47.4	<1
Marine intertidal gravel/sand beach	14.7	51
Marine intertidal mudflats	0.9	<1
Marine rocky intertidal	1.5	19
*Maritime beach*	*10.8*	*86*
*Maritime beech forest*	*0.3*	*55*
Maritime bluff	0.1	24
*Maritime dunes*	*9.3*	*84*
*Maritime freshwater interdunal swales*	*1.3*	*74*
*Maritime grassland*	*0.6*	*47*
*Maritime heathland*	*1.7*	*81*
*Maritime holly forest*	*<0.1*	*94*
*Maritime oak forest*	*3.5*	*46*
*Maritime pitch pine dune woodland*	*3.1*	*64*
*Maritime red cedar forest*	*0.3*	*17*
*Maritime shrubland*	*4.1*	*44*
Marl fen	4.7	1
Marl pond	0.7	0
Marl pond shore	<0.1	0
Marsh headwater stream	1.7	<1
Medium fen	7.7	<1
Meromictic lake	0.6	2
Mesotrophic dimictic lake	11.6	<1
Mountain fir forest	65.5	<1
Mountain spruce‐fir forest	520.3	<1
Northern white cedar rocky summit	0.3	0
Northern white cedar swamp	41.9	1
Oak openings	0.8	8
Oak‐tulip tree forest	33.4	5
Oligotrophic dimictic lake	116.5	<1
Oligotrophic pond	0.3	1
Open alpine community	1.0	0
Oxbow lake/pond	0.7	<1
Patterned peatland	1.7	0
Perched bog	<0.1	0
Perched swamp white oak swamp	0.2	0
Pine barrens shrub swamp	0.4	32
Pine barrens vernal pond	0.1	69
Pine‐northern hardwood forest	36.6	42
Pitch pine‐blueberry peat swamp	3.9	7
*Pitch pine‐heath barrens*	*16.4*	*75*
*Pitch pine‐oak forest*	*133.2*	*89*
Pitch pine‐oak‐heath rocky summit	39.3	<1
*Pitch pine‐oak‐heath woodland*	*50.1*	*84*
*Pitch pine‐scrub oak barrens*	*37.8*	*85*
Post oak‐blackjack oak barrens	<0.1	51
Red cedar rocky summit	6.6	<1
Red maple‐blackgum swamp	4.4	15
Red maple‐hardwood swamp	34.1	2
Red maple‐swamp white oak swamp	0.1	4
Red maple‐sweetgum swamp	1.6	5
Red maple‐tamarack peat swamp	11.7	<1
Red pine rocky summit	0.8	0
Rich graminoid fen	5.2	1
Rich hemlock‐hardwood peat swamp	11.7	<1
Rich mesophytic forest	161.2	<1
Rich shrub fen	2.9	<1
Rich sloping fen	0.8	<1
Riverside ice meadow	0.9	20
Riverside sand/gravel bar	1.2	6
Rocky headwater stream	2.3	2
Rocky summit grassland	4.6	0
Salt panne	40.6	1
Salt shrub	3.8	2
Saltwater tidal creek	0.3	1
Sand beach	1.5	20
Sandstone pavement barrens	22.4	2
Sea level fen	0.3	5
Sedge meadow	7.3	<1
Serpentine barrens	0.2	<1
Shale cliff and talus community	7.4	<1
Shale talus slope woodland	3.1	9
Shallow emergent marsh	28.7	<1
Shoreline outcrop	11.3	5
Shrub swamp	32.7	<1
Silver maple‐ash swamp	62.2	1
Sinkhole wetland	1.2	<1
Spruce flats	67.2	10
Spruce‐fir rocky summit	11.4	0
Spruce‐fir swamp	24.4	6
Spruce‐northern hardwood forest	133.8	11
*Successional blueberry heath*	*11.2*	*48*
Successional fern meadow	11.2	48
*Successional maritime forest*	*2.4*	*24*
Successional northern hardwoods	19.1	83
*Successional northern sandplain grassland*	*17.2*	*65*
Successional old field	0.4	7
Successional red cedar woodland	4.1	44
Successional shrubland	0.7	0
Summer‐stratified monomictic lake	169.3	0
Talus cave community	4.2	26
Terrestrial cave community	<0.1	0
Tidal river	300.5	<1
Unconfined river	3.3	<1
Vernal pool	0.2	20
Wet alvar grassland	0.7	0
Winter‐stratified monomictic lake	3.8	<1

*Note*: Ecosystems that occur primarily on deep sandy soils (Table 1) are indicated by *italics*.

We were also interested in whether our model avoided conditions that support ecosystems outside our focus ecosystem types, such as mesic forests. We did this by calculating the proportions of areas of the 147 other native ecosystem types mapped by NYNHP that occurred within our map. Our model avoided matches with a variety of ecosystems that are not known to occur on deep sandy soils (Table A1). For example, only 2% of hemlock‐northern hardwood and floodplain forests, and less than 1% of the area of Appalachian oak‐hickory, beech‐maple mesic, and chestnut oak forests occurred within our modeled area (Table A2).

**TABLE A2:** Animal and plant species that are known to inhabit pine barrens and sandplains ecosystems in New York State

Species	Common name	State/Federal status^a^	Affinity to focus ecosystems^b^	NYNHP area (ha)	Percentage match with soil model
Moths and butterflies					
*Abagrotis benjamini*	Coastal heathland cutworm moth	S1S3/G3	High	21.9	16
*Acronicta albarufa*	Barrens dagger moth	S1/G3G4	High	2.4	100
*Anisota stigma*	Spiny oakworm moth	SU/G5	Medium	0.7	58
*Apamea burgessi*	Burgess's apamea moth	SU/G4	Medium	117.0	5
*Apamea inordinata*	Irregular apamea moth	S1/GU	High	5.8	56
*Atrytonopsis hianna*	Dusted skipper butterfly	S2S3/G4G5	Medium	50.3	47
*Callophrys irus*	Frosted elfin butterfly	S1S2 (Threatened in NY)/G2G3	High	538.4	91
*Calycopis cecrops*	Red‐banded hairstreak butterfly	SU/G5	Medium	71.2	92
*Catocala herodias gerhardi*	Herodias underwing moth	S1S2 (Special Concern in NY)/G3T3	Medium	117.0	24
*Catocala jair* ssp. *2*	Jersey jair underwing moth	S1S2 (Special Concern in NY)/G4T4	High	19.7	99
*Cerma cora*	Bird dropping moth	S1S2/G3G4	Medium	1,469.4	90
*Chaetaglaea cerata*	Waxed sallow moth	S1S3/G3G4	High	85.5	89
*Chytonix sensilis*	Sensitive chytonix moth	S1S3/G4	Medium	30.1	96
*Cicinnus melsheimeri*	Melsheimer's sack bearer moth	S1/G4	High	2.3	100
*Cisthene packardii*	Packard's lichen moth	SU/G5	Low	13.8	76
*Cleora projecta*	Projecta gray moth	SU/G4	High	1.0	81
*Dargida rubripennis*	Pink streak moth	SU/G3G4	High	31.5	17
*Dasychira pinicola*	Pine tussock moth	SU/G4	High	1.4	5
*Datana ranaeceps*	A hand‐maid moth	S1S3/G3G4	High	3.1	94
*Derrima stellata*	Pink star moth	S1/G4	High	2.0	99
*Dichagyris acclivis*	Switchgrass dart moth	S2S3/G4G5	Medium	33.0	17
*Eacles imperialis imperialis*	Imperial moth	SU/G5T5	Medium	4.9	69
*Erastria coloraria*	Broad‐lined Catopyrrha	S1S2/G3G4	High	0.2	100
*Erynnis martialis*	Mottled duskywing butterfly	S1 (Special Concern in NY)/G3	Medium	153.1	99
*Euchlaena madusaria*	A geometrid moth	S1/G5	High	4.9	75
*Eucoptocnemis fimbriaris*	Fringed dart moth	S1/G4	High	30.6	21
*Euxoa pleuritica*	Fawn brown dart moth	S2S3/G4	High	4.2	81
*Euxoa violaris*	Violet dart moth	SU/G4	High	59.2	57
*Hemileuca maia maia*	Inland barrens buckmoth	S1S2 (Special Concern in NY)/G5T5	Medium	1,529.5	90
*Hemileuca maia* ssp. *5*	Coastal barrens buckmoth	S2 (Special Concern in NY)/G5T3	High	8,438.8	87
*Heterocampa varia*	Sandplain heterocampa	S1S2 (Special Concern in NY)/G3G4	High	5.6	100
*Hydraecia stramentosa*	Hairy hydraecia moth	S1S3/G4	High	3.1	0
*Hyperstrotia flaviguttata*	Yellow‐spotted graylet moth	SU/G4	High	4.7	92
*Hypomecis umbrosaria*	Umber moth	SU/G4	High	3.0	61
*Ilexia intractata*	Black‐dotted ruddy moth	S1/GNR	High	1.8	90
*Lithophane viridipallens*	Pale‐green pinion moth	S1/G5	Low	0.7	100
*Macrochilo bivittata*	Two‐striped cordgrass moth	S1S3/G3G4	Low	22.5	80
*Marimatha nigrofimbria*	Black‐bordered lemon moth	S1/G5	Medium	4.5	74
*Metalectra richardsi*	Richard's fungus moth	SU/G4	Medium	0.5	100
*Monoleuca semifascia*	Pin‐striped slug moth	S1/G4G5	High	4.0	100
*Morrisonia mucens*	Gray woodgrain moth	S1S3/G4G5	High	2.3	99
*Oligia bridghamii*	Bridgham's brockade moth	SU/G5	Medium	2.5	63
*Parasa indetermina*	Stinging rose caterpillar moth	S1/G4	Medium	2.3	51
*Plebejus melissa samuelis*	Karner blue butterfly	S1/G1G2 (NY and Federally endangered)	High	415.8	98
*Psectraglaea carnosa*	Pink sallow moth	S2/G3	Medium	13.4	97
*Renia nemoralis*	Chocolate renia moth	SU/G4	Medium	6.3	59
*Satyrium edwardsii*	Edwards' hairstreak butterfly	S3S4/G4	Medium	3,421.0	72
*Schinia spinosae*	Spinose flower moth	SU/G4	Medium	2.8	86
*Schinia tuberculum*	Golden aster flower moth	S2/G4	Medium	0.2	100
*Schizura apicalis*	Plain schizura moth	SU/G3G4	High	1.6	99
*Speranza exonerata*	Barrens itame moth	S1S3/G3G4	Medium	96.3	7
*Sphinx gordius*	Gordian sphynx moth	S1S3/G4G5	High	2.9	80
*Sympistis perscripta*	Scribbled sallow moth	S1/G4	Medium	2.7	24
*Sympistis riparia*	Dune sympistis moth	SU/G4	Medium	11.6	67
*Virbia aurantiaca*	Orange Holomelina moth	SU/G5	Medium	6.1	73
*Zale lunifera*	Pine barrens zale moth	SU/G3G4	High	3.2	100
*Zanclognatha martha*	Pine barrens zanclognatha moth	S1S2/G4	Medium	33.5	86
			Area weighted average = 83%		
Vertebrates					
*Scaphiopus holbrookii*	Eastern spadefoot toad	S2S3 (Special Concern in NY)/G5	Medium	2358.4	79
Plants					
*Carex houghtoniana*	Houghton's sedge	S2 (Threatened in NY)/G5	Medium	10.5	51
*Cyperus schweinitzii*	Shweinitz's flat sedge	S3 (Rare in NY)/G5	High	80.5	80
*Desmodium ciliare*	Hairy Small‐Leaved Ticktrefoil	S2S3 (Threatened in NY)/G5	Medium	25.4	38
*Lupinus perennis*	Wild blue lupine	S3 (Rare in NY)/G5	Medium	54^c^	72
*Viola pedata*	Bird's‐foot violet	S2 (Threatened in NY)/G5	Medium	9^c^	56

^a^ New York State conservation rankings, S1 to S5, where S1 is for the most imperiled species and S5 for species that are demonstrably secure in the state. SU is for species that are unranked. Global conservation rankings, G1 to G5, where G1 is for critically imperiled species to G5 for species that are globally secure. Even species with a global ranking of G5 may be rare in parts of their ranges. Where a ranking straddles two categories (e.g., S2S3), there is not enough information to distinguish between ranks. Some species are also recognized as threatened, endangered, “special status species,” or rare in NY State and the US. Special status species are not yet recognized as threatened or endangered, but documented evidence exists that their continued existence in New York is imperiled.

^b^ Each species' affinity to the focus ecosystems (Table 1) versus other kinds of ecosystems (including rock outcrops, mesic forests, and disturbed sites)

^c^ Location points.
